# Supplementary material for: Rectal swabs vs bulk faeces PCR testing for the diagnosis of enteric conditions (RecSwabFaeces): Protocol for a single group diagnostic accuracy comparative trial
Source: PLoS One. 2025 Aug 22;20(8):e0329643. doi: 10.1371/journal.pone.0329643 (PMC12373177; doi:10.1371/journal.pone.0329643)
Supplement: S2 File — (DOCX) [file pone.0329643.s002.docx]

Rectal Swabs vs Bulk Faeces PCR testing for the Diagnosis of Enteric Conditions (RecSwabFaeces)

Principal Investigator: Smoll NR,

Chief Investigators: Williams D, Khandaker G, Subedi S, Schlebusch S, Thomas M

Study Nurses: Neucom D, Barnekow D, Walker J

# Summary

This grant proposal seeks funding to investigate whether rectal swabs are a reliable proxy for fecal sampling. This would possibly revolutionise daily clinical practice, allowing for faster specimen collection, improving the patient’s testing experience, and increasing the speed and precision care can be delivered. The proposed study will compare paired stool and rectal swab sample PCR testing in a variety of settings, including suspected norovirus clusters, Hepatitis A clusters, hospital and community settings.The sample size is expected to be less than 600, and the statistical analysis will use 2x2 tables to evaluate the sensitivity, specificity, positive and negative predictive values against the gold standard of bulk faeces testing.

Consumer engagement and the dissemination of results will be a key part of the study. The researchers will work closely with the Sunshine Coast Health Consumer Research Engagement Group, through several formal and informal channels. Because the research is intended to change clinical practice, an extensive dissemination plan is to be enacted across the nation, and internationally.

Contents

[Summary 1](#_Toc141712115)

[Plain Language Statement 2](#_Toc141712116)

[Introduction 2](#_Toc141712117)

[Aims and Outcomes 3](#_Toc141712118)

[Methods 4](#_Toc141712119)

[Timeline 4](#_Toc141712120)

[Sample Size 5](#_Toc141712121)

[Recruitment 7](#_Toc141712122)

[Consent Process 8](#_Toc141712123)

[Statistical Analysis 8](#_Toc141712124)

[Consumer Engagement Plan 9](#_Toc141712125)

[Dissemination Plan 9](#_Toc141712126)

[Impact 9](#_Toc141712127)

[Strategic Objective Alignments 10](#_Toc141712128)

[SCHHS Strategic Plan 2022-2026 10](#_Toc141712129)

[Sunshine Coast Public Health Unit Research Strategic Plan 2023-2025 10](#_Toc141712130)

[References 10](#_Toc141712131)

# Plain Language Statement

Obtaining a stool specimen for testing can be challenging and an unpleasant experience. A person is required to obtain a sample pot from a healthcare location, return home, wait until they have a bowel motion and then work out how to collect the sample during or after passing the stools, and then transport it back to the clinic. If after-hours they have to also keep the sample in their house (possibly in the fridge) to transport it when the clinic is open. In the hospital, a person must wait in their room until they need to have a bowel motion, then go to the bathroom, and then collect the sample themselves. We propose a much simpler method, allowing the person to do the sampling “on-demand” or in the comfort of their own home.

Collecting a swab from the anorectum would allow the person to test themselves, “on-demand” by simply inserting a swab into their rectum. This is similar to self-testing for human papilloma virus, within the cervical cancer screening program.

Here, we will attempt to confirm the validity and reliability of a rectal swab self-collection to see if it provides the same results as bulk faeces testing.

This would possibly revolutionise daily clinical practice, allowing for faster specimen collection, improving the patient’s testing experience, and increasing the speed and precision care can be delivered.

# Introduction

At present, testing for hepatitis A virus in humans necessitates the provision of a faecal sample. This method is not without drawbacks; in particular, the very nature of defecation means that samples cannot be provided ‘on demand’ in a physician’s office or to a research nurse and are reliant on appropriate collection by patients themselves.^1^ Patients in hospitals with diarrhoea commonly flush the toilet before healthcare workers can take a stool sample. Faeces sampling may also present additional complexities, including the logistical challenges of having to transport samples between patient’s home, the clinic and the laboratory, and considerable patient reluctance to providing samples. Rectal swabbing has been shown to be a valid alternative in several research and clinical settings.^2,3^

Rectal swabbing is standard practice in Australia and overseas for the surveillance and screening of multi-resistant organisms. National U.K and Australian guidance mandates the use of rectal swabs for screening for intestinal colonization with carbapenemase-producing Enterobacteriaceae for at-risk patients admitted to healthcare settings.^3,4^ The key benefits of rectal swabs include the ease with which they can be administered and transported, and the high levels of acceptability to patients/research study participants.^3^

Self-collection as a process has been shown to be an accurate testing methodology in other settings. Human papilloma virus self-collection has been shown to be just as accurate, or even more sensitive than clinician collected samples.^5^ The accuracy of rectal swabs vs bulk faeces testing has been tested extensively using culture mediums, but much less work has been done since the advent of PCR testing methodology.

Studies have long compared rectal swabs vs bulk faeces testing. The results are varied, depending on the year the study was performed. An early study examining 700 specimens tested with MacConkey's medium in 1940 during a Sonne dysentery outbreak (Shigella sp. outbreak) found that rectal swabbing was far superior to bulk faeces culture to detect an organism.^6^ Rectal swabbing became the standard for a period of time This was disputed later in 1954 after a group examined 3,183 index cases looking for a variety of organisms to which they concluded that the *“…626 [bulk faeces] specimens all showed abnormalities which could be demonstrated only by microscopy or simple chemical methods, for which the rectal swab is quite unsuitable”.*^7^ Since then, huge changes have occurred to our detection methods (e.g. culture mediums) and the advent of PCR testing for multiple organisms at once.

More recent studies demonstrate that the differences between rectal and faecal swabs is driven by the type of test (bacterial microscopy and culture vs viral PCR). During a phase I clinical study for a live oral attenuated Salmonella Typhi vaccine strain found that when compared with fecal culture, rectal swabs were 64% sensitive and 90% specific.^8^ A recent study on paired samples found that for bacteria, the sensitivity was 86.5% (95% CI 79.5%, 91.8%) when PCR was performed and 61.4% (95% CI 52.4%, 69.9%) when culture for bacteria was performed, indicating better performance for PCR.^9^

It is has been shown in small studies that rectal swabs are likely accurate. Studies are have now shown that during testing for various enteric organisms (viruses and bacteria) in Rwandan children there was no significant difference in detection rate between faeces and rectal swabs for any agent, reflecting that pathogen concentration was far above the limit of detection in the majority of cases.^10^ A similar paired sample study focusing on Norovirus found a sensitivity of rectal swabs that was greater than 97%, and in one case detected norovirus in the swab, but not in the bulk faeces.^11^

The outcome of this project will transform clinical practice for doctors and patients. It will make testing far more convenient, and allow for point-of-care testing to become a reality for enteric organisms. Currently, many people leave the emergency department without a faecal test because they could not produce a specimen while they are with the clinician. Rectal swabbing would make the diagnosis, and better care available for these patients. Public Health practitioners would be able to perform mass testing after small or large scale outbreaks, such as those caused by hepatitis A, norovirus or other organisms found in faeces.^12,13^ Doctor’s in the emergency department would be able to obtain swabs on demand for testing that would expedite time spent in ED and with the earlier diagnosis, length of stay in hospital. Most importantly, for patients, it means that they no longer must go home, wait until they need to poo, and then return to the hospital or GP clinic with their sample, as it can be done on demand, on-site (in a private situation). Thus, rectal swabbing can transform clinical practice through earlier diagnosis, shorter length of stay’s and greatly improving the patient experience.

# Aims and Outcomes

This study aims to demonstrate that self-collected or clinician collected rectal swabs are a valid alternative method of testing when compared to bulk faeces testing for a wide range of enteric organisms such as bacteria, viruses and parasitic/protozoans. The intended outcome of this project is to replace bulk faeces testing with rectal swabbing in daily clinical and public health practice.

# Methods

We will compare paired stool and rectal swab sample PCR testing to investigate whether rectal swabs are a reliable proxy for faecal sampling. Tests would be done in accordance with normal clinical practice. Persons who would normally be provided with a faecal specimen jar, will also be provided with a rectal swab to perform themselves (within 24 hours of the faeces collection, preferably as the first step). The person will obtain 1x faecal sample and 1x rectal swab sample. Rectal swab collection was carried out at the same time as stool sample production and was obtained by self-insertion via the anus to a depth of 2–3 cm and rotated 3-5 times and ensure it is soiled. Faecal samples and rectal swabs will be returned to a local hospital to be sent to Pathology Queensland. These will be sent to FSS for paired sample testing of Hepatitis A PCR if they are part of a Hepatitis A cluster. The samples will be sent to Pathology Queensland for multiplex PCR assays for a range of organisms (including norovirus) if the setting is in community settings or hospital and not part of an outbreak.

**Organisms to be tested for by Pathology Queensland:**

**GI-Bacteria(I) Assay** (hereafter called ‘Bacteria Panel I’)

Shigella spp./ Enteroinvasive Escherichia coli (Sh/EI), Campylobacter spp. (CAM), Yersinia enterocolitica (YER). Vibrio spp. (VIB), Clostridium difficile toxin B (CdB), Aeromonas spp. (AER), Salmonella spp. (SAL)

**GI-Virus Assay** (hereafter called ‘Virus Panel’):

Norovirus genotype I (NVG1), Norovirus genotype II (NVG2), Rotavirus A (ROV), Adenovirus F (Serotype 40/41) (ADV-F), Astrovirus (ASV), Sapovirus (Genogroups G1, 2, 4, 5) (SV)

**GI-Parasite Assay** (hereafter called ‘Parasite Panel’):

Giardia lamblia (GL), Entamoeba histolytica (EH), Cryptosporidium spp. (CR), Blastocystis hominis (BH), Dientamoeba fragilis (DF), Cyclospora cayetanensis (CC)

## Test Transport, Storage and Results

The bulk faeces specimen and the rectal swab will be transported at 4°C and be retained for minimum 1 month as per standard laboratory protocols for such materials. A small sample (aliquot) of the bulk faeces specimen and the rectal swab will be retained for minimum 1 year after submission, or until the end of the project (whichever is later).

The bulk faeces test will be the test that will be reported on for positive or negative test results. For samples that are discordant (e.g. swab negative but bulk faeces positive for organism, or vice versa), the bulk faeces will be deemed the accurate test. Since bulk faeces is amenable to retesting, the sample will be retested if the bulk faeces is negative and the swab is positive for the organism.

The treating clinician will be blinded to the results of the swab. However, the treating clinician will not be blinded to the bulk faeces test and will be responsible for reporting the result to the patient.

## Timeline

The expectations are that we will obtain an average of 3-6 swabs per week by public health nurses. Some situations, such as outbreaks or mass gathering events will allow for greater numbers of swabs to be collected in a small timeframe. Outside of these events, paired testing will occur in the hospital, in collaboration with our Infectious Diseases team. For example, if a patient has been identified on the ward of needing a sample, the study nurse would ensure the appropriate channels are followed: consenting the patient, ensuring the right samples are obtained, and ensuring their transport to the appropriate lab for testing.

- Year 1 (Q1 & Q2) – Administration and onboarding, ethics and site specific agreements. Expecting a maximum of 50 paired samples (approx. 2/week). Interim analysis #1 for completed samples. Consumer engagement meeting #1.
- Year 1 (Q3 & Q4) – Interim analysis and quality review to be translated to research team practice and crossing of the 100 sample milestone (reach 4 samples/week).
- Year 2 (Q1 & Q2) – Reach 6 samples per week on average cross the 250 sample milestone. Interim analysis #2 and quality review to be translated to research team practice. Consumer engagement meeting #2, and ethics report.
- Year 2 (Q3 & Q4) – Continue 6 samples per week on average and cross the 400 sample milestone.
- Year 3 (Q1 & Q2) – Continue 6 samples per week, reaching up to 550-600 samples. Aim for hard closure of study by the end of Q2, to provide lag time for lab testing to occur.
- Year 3 (Q3 & Q4) – Feedback to consumer engagement team, final analyses and report, and dissemination plan including media releases, travel to key conferences (e.g. CDIC, RACP/RACS meetings, Surgical/Medical conferences. The final publication will be aimed for the Medical Journal of Australia.

### Milestones

Milestone 1. Ethics, onboarding research coordinator and consumer engagement group #1. Month 1-6

Milestone 2. First 50 samples with interim analysis and quality assurance translated to practice. Month 3-6

Milestone 3. Average 6 samples taken per week. Month 9-12

Milestone 4. First 250 samples taken, interim analysis #2 completed. Month 18-24

Milestone 5. Over 400 samples taken. Month 30

Milestone 6. Final report written and submitted to major journal

## Sample Size

A sample size analyses with a wide range of input parameters (see Figure 1), demonstrates that less than 600 samples will be sufficient. If we assume that 35% of samples will contain an organism (“proportion positive”) with expecting a sensitivity of at least 95% (“sensitivity priors”) means we will require a sample size of no more than 300 paired samples.

The smaller the resources that the team has (e.g. funding), the higher the pre-test probability at the time of test must be to estimate with confidence (see figure 1). For example, if we only have funding for 40 persons tested, we would elect to test persons with clear known linkages to a person with the illness (symptomatic family member of someone diagnosed with norovirus), or we would perform clearance testing in persons with known disease using these methods (the guidelines call for clearance testing for a variety of illnesses). If we have more funding, testing can approximate normal clinical encounters and have less restrictive criteria for testing. If the funding is limited to 40 samples, we will endeavour to control the testing method, aiming for in-clinic self-testing.


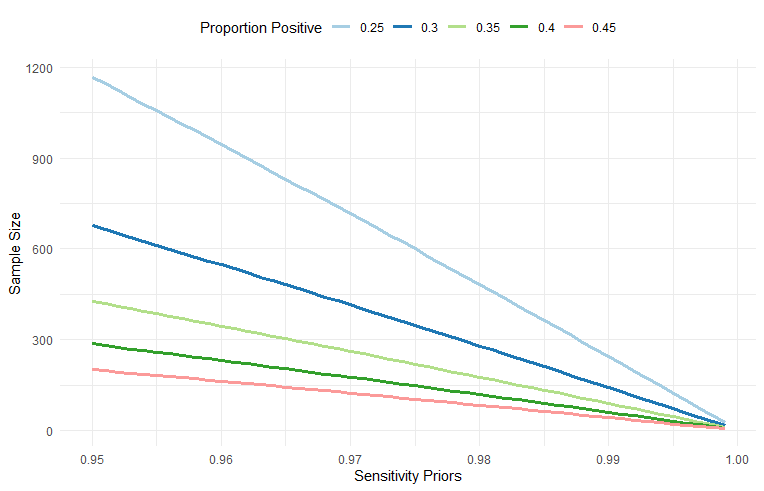


**Figure 1. Sample size analysis** using a range of parameters. The “Sensitivity priors” represents the approximate expected sensitivity of our method, and the “proportion positive” represents the proportion of samples that will contain an organism, which also means that should test positive.


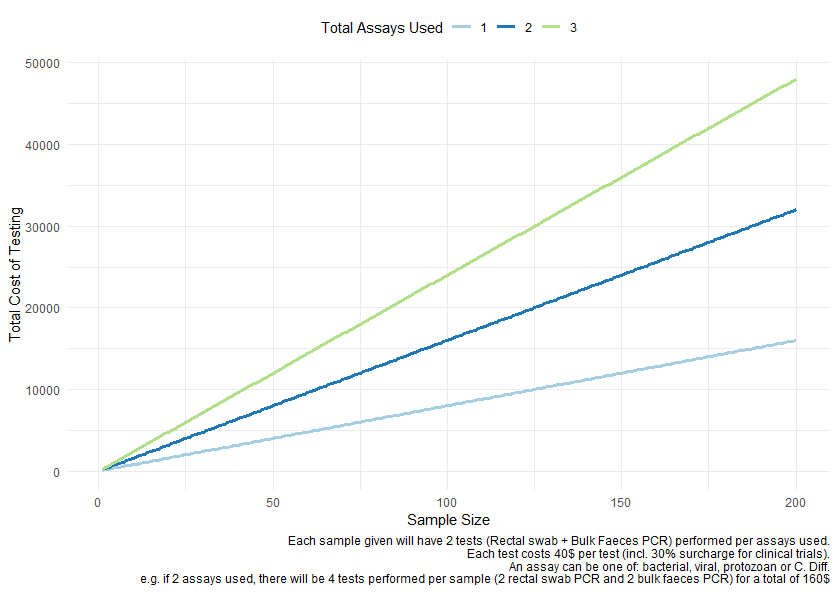


**Figure 2.** Cost modelling for the project. Ideally, only one assay will be needed to be used. No cost buffers included.

## Recruitment

There are only two identified regions for recruiting participants, the Sunshine Coast Hospital and Health Service and the Central Queensland Hospital and Health Service.

The recruitment strategy will be multifaceted to obtain samples from a range of representative clinical settings, e.g. outbreaks, community and in-hospital. The strategy will involve a central study coordinator supporting acquisition of samples, coordinating the multiple sources. For outbreaks, the study coordinator will be able to send via express post, samples to the patient, family, and/or symptomatic members of an outbreak. In community settings, the study coordinator can obtain samples from GP practices that are seeing patients recently seen as inpatients in hospital – identified from admission lists, or our Infectious Diseases Physician study collaborators. Inpatients, especially those which represent persons with a high pre-test probability, will be identified, recruited via their treating practitioner, and then consented to take part in the study. Consent will be performed verbally over the phone by a study coordinator (records kept locally), and/or with a physical form sent in a return envelope.

Inclusion criteria includes:

- Persons aged 18 and older
- Able to consent to and perform the additional rectal swab test.
- Close contact or person exposed, suspected or confirmed case associated with an outbreak of an enteric organism. E.g. hepatitis A outbreak, foodbourne outbreak.
- Chronic carrier of an enteric organism that requires testing.
- Person involved in a clinical encounter where they are, or likely suffering from a disease caused by an enteric organism. (E.g. person presenting to the GP or Emergency Department with diarrhoea or jaundice)

Exclusion Criteria

- Persons unlikely (as judged by the clinician) to be harbouring an enteric organism.

## Follow-up

Each person will receive a follow-up phone call after the test has been completed. This is as part of usual clinical practice, but we will ask if the person:

1. Had an adverse event during or after the testing, and the nature of the event.
2. Which test did you prefer? And follow-up with why.

## Consent Process

In all study settings, the potential participant will be asked by the treating clinician for consent to be contacted by the study coordinator. After which the study coordinator will contact the person to obtain consent.

There will be two ways that a participant can consent to participating (verbal, written). Verbal consent will be obtained over the phone with a study coordinator or study nurse, as is commonly done in outbreak situations. The study nurse will read the patient information sheet to the participant over the phone, ensures the participant understands the process and will document this in a database held on Queensland Health servers. The testing pack will be sent to the participant and will include the patient information sheet about the study and all contact details for questions about the study. Furthermore, implicit consent will occur when the participant obtains both samples (the participant can decline to participate by not providing the rectal swab) and returns one or both samples to the pathology laboratory.

If the testing is done in person (a study clinician is present), verbal, written and implicit consent (the patient will obtain the sample themselves and return it to the study coordinator) will be obtained using the patient information sheet, signature document kept, with a copy offered to the participant.

Opting out of the study will be possible for participants at all stages of the study, up until the analysis of the research. For example, a participant may agree to participation over the phone, then, once they receive the full pack with the Patient Information Sheet, and they read over it in detail, they can withdraw from the study by calling us and withdrawing, or, not sending the rectal swab, and then withdrawing after we check in with them after the rectal swab was not returned.

### Risks and Harm

The key concerning harm is accidental stigmatization. Stigmatization can occur if someone accidentally assumes that the rectal swabbing is for a sexually transmitted infection of the buttock. Rectal swabs can be used in rare situations to swab for sexually transmitted infections acquired during anal intercourse. The risk of this is quite low, even without safeguards in place as few people, even in the health community know that rectal swabs are often used for sexually acquired infections. To safeguard against this, the swabs will be given or sent to the participant in opaque envelopes, and the pathology forms will be labelled appropriately with correct symptomatology in the “clinical notes” section i.e. “gastroenteritis/diarrhoea/suspected foodborne illness/Hepatitis A outbreak etc”, as appropriate.

## Data storage, access and de-identification

This study will collect data related to routine clinical practice (bulk faeces testing), as well as research data (rectal swab results). The results of these tests will be kept in a clinical database (AUSLAB, within Queensland Pathology) as well as within local study databases (Excel spreadsheets or Redcap Databases) for operational and analytical purposes. At all times, data will be kept within Queensland Health databases and will not be shared with external entities. Only Queensland Health staff (includes Pathology Queensland, and Forensic Scientific Services) will have access to the clinical and research data.

Data will be de-identified prior to proceeding to the statistical analysis in accordance with Section 164.514(a) of the HIPAA Privacy Rule provides the standard for de-identification of protected health information, the Australian De‐Identification Decision‐Making Framework, and AIHW guidelines for disclosure of secondary use health information and the Australian Privacy Act and Australian Privacy Principles.^14–17^

### Deidentification Methods

# De-identification involves two steps. The first is the removal of direct identifiers. The second is taking one or both of the following additional steps: 1) the removal or alteration of other information that could potentially be used to re-identify an individual, and/or 2) the use of controls and safeguards in the data access environment to prevent re-identification.^18^ The study data will be de-identified using the following methods:

# Direct identifiers, such as names, addresses, will be removed as soon as the data integrity is verified and cleaned and prior to analysis, and never reported publicly.

# Indirect identifiers, such as dates of birth, zip codes, and medical codes, will be removed as soon as the data integrity is verified and cleaned and prior to analysis, and never reported publicly.

- Removal of the direct and indirect identifiers are done as early as possible in the data cleaning phase to safeguard the data in case of re-identification.

# When presented or published, data is *always aggregated and grouped* so that identification is not possible. We will suppress cells/rows or information with 5 or less persons and avoid reporting outliers in ranges (e.g. such as persons aged 107 years of age, or a death in a 2 month old).

### Public Release Model

The public release modelled data occurs after extensive cleaning and analysis. The released data is *always* aggregated for descriptive analyses. The focus of the analysis is mathematical models that provides only group-wide estimates. For example, mathematical models report odds/hazard ratios, relative risks or effectiveness estimates. In both situations, re-identification remains impossible.

## Statistical Analysis

We will assess the sensitivity, specificity, positive predictive value, negative predictive value using 2x2 tables. The gold standard will be bulk faeces PCR testing. Sensitivity is the proportion of individuals with the disease who test positive with rectal swabs. Specificity is the proportion of individuals without the disease who test negative with rectal swabs. Sensitivity = True positives / (True positives + False negatives). Specificity = True negatives / (True negatives + False positives). We will assess the cycle threshold values using general linear models if the relationship is linear as expected.

The McNemar test is a non-parametric test used to analyse paired nominal data. It is a test on a 2 x 2 contingency table and checks the marginal homogeneity of two dichotomous variables. The test requires one nominal variable with two categories (dichotomous; disease or no disease) and one independent variable with two dependent groups (rectal swab vs bulk faeces). The minimal sample size required for the McNemar test is at least ten discordant pairs. It should be noted that if the sum of discordant pairs is small (<25), even if the total sample size is large, the statistical power of the McNemar test will be low and the exact binomial test can be used. All results will be reported using 95% confidence intervals.

The R statistical suite will be used for all analyses and data visualisation. All estimates will be presented with 95% confidence intervals (CI). P-values <0.05 are deemed significant, and p-values <0.1 are considered trends.

## Consumer Engagement Plan

Working closely with the Sunshine Coast Health Consumer Research Engagement Group is a key part of this project. We will aim to attend formal consumer group meetings 1-2x/year, and work with consumers in various informal settings. The aims of our engagement are to engage consumers in the research process and obtain their feedback on the new method of collecting stool specimens, to educate consumers about the benefits of the new method and how it could improve their healthcare experience. Lastly, this plan will support the dissemination of information to build support for the new method among consumers and healthcare providers.

## Dissemination Plan

We will aim for a comprehensive global dissemination plan, starting locally and progressing to nationally across a wide range media. Information will be released to the local news networks (7, 9 and 10, ABC and SBS), as well as radio interviews will be offered to ensure we engage the general public. I will aim to provide Grand rounds talks to multiple major hospitals inside and outside of Queensland (e.g. Royal Brisbane Hospital. Royal Melbourne Hospital, Liverpool Hospital in Sydney, Royal Perth Hospital), and key pathology laboratories (QML, SnP, Queensland Pathology, Dorevitch Victoria). There will be travel included to key conferences e.g. CDIC, RACP/RACS meetings, Surgical/Medical conferences to maximise in-person communication to clinicians and academics. The final publication will be aimed for the Medical Journal of Australia. Overall, this dissemination plan will reach consumers and general public, the clinicians that will treat them, the pathologists that will perform the test and the academics that may use this in their future work.

# Impact

The proposed research hypothesises that this new method for collecting stool specimens is just as accurate as bulk faeces testing but is more convenient and comfortable for patients. This novel methodology would involve collecting a swab from the anorectum, which could be done at home or in a healthcare setting. This would allow patients to collect the sample when it is convenient for them, and it would eliminate the need to transport the sample to a clinic.

These changes to testing would have a number of positive impacts. First, it would improve the patient experience by making the process of collecting a stool specimen more convenient and comfortable. Second, it would increase the speed and accuracy of diagnosis by allowing patients to collect the sample when it is freshest. Third, it would reduce the cost of healthcare by eliminating the need for patients to travel to a clinic to collect a stool specimen. Fourth, the ease of testing would mean higher testing rates leading to better diagnoses, and more accurate clinical care.

Overall, the development of this new method of collecting stool specimens would have a significant positive impact on patients, healthcare providers, and the healthcare system as a whole. The research project described in this impact statement has the potential to revolutionize daily clinical practice by making the process of collecting stool specimens more convenient, comfortable, and accurate.

# Strategic Objective Alignments

## SCHHS Strategic Plan 2022-2026

### Our Care

- Improve access to services and grow into our tertiary provider status in line with our Master Clinical Service Plan.
- Safeguard our community with our pandemic and disaster preparedness and response.

### Our Sustainability

- Transform non-admitted care to improve patient outcomes.

### Our Future

- Integrate research, education and clinical care in collaboration with Sunshine Coast Health Institute and other partners.

## Sunshine Coast Public Health Unit Research Strategic Plan 2023-2025

### Notifiable conditions prevention and control

This project will support the timely diagnosis of notifiable conditions thus improving surveillance. In addition, it will support the patient experience for those involved.

# References

1. Lecky DM, Hawking MK, McNulty CA. Patients’ perspectives on providing a stool sample to their GP: a qualitative study. Br J Gen Pract. 2014 Nov;64(628):e684–93.

2. Radhakrishnan ST, Gallagher KI, Mullish BH, Serrano-Contreras JI, Alexander JL, Miguens Blanco J, et al. Rectal swabs as a viable alternative to faecal sampling for the analysis of gut microbiota functionality and composition. Sci Rep. 2023 Jan 10;13(1):493.

3. Currie K, King C, McAloney-Kocaman K, Roberts NJ, MacDonald J, Dickson A, et al. The acceptability of screening for Carbapenemase Producing Enterobacteriaceae (CPE): cross-sectional survey of nursing staff and the general publics’ perceptions. Antimicrob Resist Infect Control. 2018 Dec;7(1):144.

4. National Health and Medical Research Council. Australian Guidelines for the Prevention and Control of Infection in Healthcare (2019) [Internet]. Canberra: Commonwealth of Australia; 2019 [cited 2023 Aug 29]. Available from: https://www.safetyandquality.gov.au/sites/default/files/2021-02/australian_guidelines_for_the_prevention_and_control_of_infection_in_health_care_-_current_version_-_v11.4_5_january_2021.pdf

5. Snijders PJF, Verhoef VMJ, Arbyn M, Ogilvie G, Minozzi S, Banzi R, et al. High-risk HPV testing on self-sampled *versus* clinician-collected specimens: A review on the clinical accuracy and impact on population attendance in cervical cancer screening. Int J Cancer. 2013 May 15;132(10):2223–36.

6. Cruickshank R, Swyer R. An Outbreak of Sonne Dysentery. The Lancet. 1940 Dec;236(6122):803–5.

7. Thomas MEM. Disadvantages of the Rectal Swab in Diagnosis of Diarrhoea. Br Med J. 1954 Aug 14;2(4884):394–6.

8. Kotton CN, Lankowski AJ, Hohmann EL. Comparison of rectal swabs with fecal cultures for detection of Salmonella typhimurium in adult volunteers. Diagn Microbiol Infect Dis. 2006 Oct;56(2):123–6.

9. Kotar T, Pirš M, Steyer A, Cerar T, Šoba B, Skvarc M, et al. Evaluation of rectal swab use for the determination of enteric pathogens: a prospective study of diarrhoea in adults. Clin Microbiol Infect. 2019 Jun;25(6):733–8.

10. Kabayiza JC, Andersson ME, Welinder-Olsson C, Bergström T, Muhirwa G, Lindh M. Comparison of rectal swabs and faeces for real-time PCR detection of enteric agents in Rwandan children with gastroenteritis. BMC Infect Dis. 2013 Sep 27;13:447.

11. Sidler JA, Käch R, Noppen C, Dangel M, Battegay M, Bingisser R, et al. Rectal swab for detection of norovirus by real-time PCR: similar sensitivity compared to faecal specimens. Clin Microbiol Infect. 2014 Dec;20(12):O1017–9.

12. Franklin N, Camphor H, Wright R, Stafford R, Glasgow K, Sheppeard V. Outbreak of hepatitis A genotype IB in Australia associated with imported frozen pomegranate arils. Epidemiol Infect. 2019 Jan 25;147:e74.

13. Kwon SY, Park SH, Yeon JE, Jeong SH, Kwon OS, Lee JW, et al. Clinical Characteristics and Outcomes of Acute Hepatitis A in Korea: A Nationwide Multicenter Study. J Korean Med Sci. 2014 Feb;29(2):248–53.

14. Office for Civil Rights. Guidance Regarding Methods for De-identification of Protected Health Information in Accordance with the Health Insurance Portability and Accountability Act (HIPAA) Privacy Rule [Internet]. HHS.gov. 2012 [cited 2023 Jul 13]. Available from: https://www.hhs.gov/hipaa/for-professionals/privacy/special-topics/de-identification/index.html

15. OAIC. Guide to data analytics and the Australian Privacy Principles [Internet]. OAIC. 2023 [cited 2023 Jul 13]. Available from: https://www.oaic.gov.au/privacy/privacy-guidance-for-organisations-and-government-agencies/more-guidance/guide-to-data-analytics-and-the-australian-privacy-principles

16. Office of the Australian Information Commissioner. De-identification and the Privacy Act [Internet]. Australian Government; 2018 Mar. Available from: oaic.gov.au

17. Information and Privacy Commisioner of Ontario. De-identification Guidelines for Structured Data [Internet]. [cited 2023 Jul 13]. Available from: https://www.ipc.on.ca/wp-content/uploads/2016/08/Deidentification-Guidelines-for-Structured-Data.pdf

18. O’Keefe C, Otorepec S, Elliot M, Mackey E, O’Hara K. The De‐Identification Decision‐Making Framework. CSIRO Rep [Internet]. 2017 Sep 18 [cited 2023 Jul 13];EP173122 and EP175702. Available from: https://publications.csiro.au/publications/publication/PIcsiro:EP173122
